# Why people engage in corrupt collaboration: an observation at the multi-brain level

**DOI:** 10.1093/cercor/bhad132

**Published:** 2023-04-20

**Authors:** Dandan Zhang, Shen Zhang, Zhen Lei, Yiwei Li, Xianchun Li, Ruolei Gu

**Affiliations:** Institute of Brain and Psychological Sciences, Sichuan Normal University, Chengdu 610066, China; China Center for Behavioral Economics and Finance & School of Economics, Southwestern University of Finance and Economics, Chengdu 611130, China; School of Biological Science and Medical Engineering, Beihang University, Beijing 100191, China; China Center for Behavioral Economics and Finance & School of Economics, Southwestern University of Finance and Economics, Chengdu 611130, China; Institute of Brain and Psychological Sciences, Sichuan Normal University, Chengdu 610066, China; School of Psychology and Cognitive Science, East China Normal University, Shanghai 200062, China; CAS Key Laboratory of Behavioral Science, Institute of Psychology, Beijing 100101, China; Department of Psychology, University of Chinese Academy of Sciences, Beijing 100049, China

**Keywords:** corrupt collaboration, functional near-infrared spectroscopy, hyperscanning, dorsolateral prefrontal cortex, temporal–parietal junction

## Abstract

Recent studies suggest that corrupt collaboration (i.e. acquiring private benefits with joint immoral acts) represents a dilemma between the honesty and reciprocity norms. In this study, we asked pairs of participants (labeled as A and B) to individually toss a coin and report their outcomes; their collective benefit could be maximized by dishonestly reporting (a corrupt behavior). As expected, the likelihood of corrupt behavior was high; this probability was negatively correlated with player A’s moral judgment ability but positively correlated with player B’s empathic concern (EC). Functional near-infrared spectroscopy data revealed that the brain-to-brain synchronization in the right dorsolateral prefrontal cortex was associated with fewer corrupt behaviors, and that it mediated the relationship between player A’s moral judgment ability and corrupt collaboration. Meanwhile, the right temporal–parietal junction synchronization was associated with more corrupt behaviors, and that it mediated the relationship between player B’s EC and corrupt collaboration. The roles of these 2 regions are interpreted according to the influence of the honesty and reciprocity norms on corrupt collaboration. In our opinion, these findings provide insight into the underlying mechanisms and modulating factors of corrupt collaboration.

## Introduction

Corrupt collaboration refers to the phenomenon that 2 or more individuals act together toward a mutually beneficial outcome through unethical means (Weisel and Shalvi 2015; Kobis et al. 2021; Leib et al. 2021). Corrupt collaboration represents in many forms, from theft and embezzlement in local business units to systematic and organizational corruption in transnational entities (Gaspar and Hagan 2016). As a notorious phenomenon in modern days, corrupt collaboration has been producing adverse social and economic outcomes around the world and is becoming a threat to the stability of societies and to an honest culture (Adegbite et al. 2012; Köbis et al. 2016, 2017; Wachs et al. 2019; Gorsira et al. 2020). Although corrupt collaboration has been the focus of numerous economic, sociological, and organizational studies, a basic question remains largely unresolved in the literature: why are many people corrupt each other and become partners in crime, regardless of its unethical nature and legal consequence? An intuitive, utility-based explanation is that the potential benefits of corrupt collaboration are worth the risk from an individual’s perspective (Aguilera and Vadera 2008; Tavits 2010). Nevertheless, this hypothesis has turned out to be unsatisfactory across many situations, since in these situations corrupt behavior is actually not driven by cost-benefit calculation (Rosenblatt 2002; Ashforth et al. 2008; Zaloznaya 2012).

To address the above issue, Weisel and Shalvi (2022) recently interpret corrupt collaboration as a tension between 2 competing motives: to be honest or to build up a reciprocal relationship with others (Gneezy 2005; Haidt 2007; Gross et al. 2018). Both honesty and reciprocity are basic social norms in human life (Gintis et al. 2008; Abeler et al. 2019). On the one hand, honesty is regarded by almost all human societies as a crucial moral value (Gächter and Schulz 2016), to the extent that honest behavior is intuitive and automatic for most individuals (Lundquist et al. 2009; Köbis et al. 2019). On the other hand, human beings are prone to cooperate with other individuals (including unrelated ones) under the reciprocity norm, that is, returning favors and other acts of kindness (Gouldner 1960; Nowak 2006). Following the reciprocity norm could justify unethical practices and free people from moral restraint (Cohen et al. 2009; Lambsdorff and Frank 2011; Gino et al. 2013). Indeed, although dishonest behavior may increase psychological burden and bring about negative emotional reactions (e.g. guilty, cynicism, pessimism, or paranoia), such outcomes could be alleviated when not only oneself but also other persons are benefited from dishonesty (Pelletier and Bligh 2008; Wiltermuth 2011; Conrads et al. 2013; Gino et al. 2013; Shalvi et al. 2015). According to Weisel and Shalvi (2022), when honesty and reciprocity are in conflict, corrupt collaborations would arise if people prioritize reciprocity over honesty (see also Gneezy 2005; Levine and Schweitzer 2014; Gross et al. 2018). Furthermore, both honesty and reciprocity could diffuse among the members of an organization, because many people tend to conform to other group members who obey the honesty/reciprocity norm (Brunner and Ostermaier 2019; Isler and Gächter 2022). Thus, individuals who belong to a community or an organization with a truth-telling tradition are more likely to be honest even though it is effortful and costly (Dyreng et al. 2012; Vadi and Vissak 2013; Andrighetto et al. 2016; Derfler-Rozin and Park 2022). In the same vein, people are generally more prone to cooperate with others who are willing to reciprocate (Gächter 2006; Simpson 2007; Zhang et al. 2019). Considering that both honesty and reciprocity are “contagious,” we believe that investigating how these 2 social norms conflict during social interaction would be critical to understand the occurrence and development of corrupt collaboration.

While most studies exploring corrupt collaboration have relied on surveys and economic data sets (e.g. Adegbite et al. 2012; Langbein and Sanabria 2013; Wachs et al. 2019; Gorsira et al. 2020), a recent empirical study conducted by Weisel and Shalvi (2015) successfully captured the phenomenon of corrupt collaboration in behavioral laboratory for the first time. Specifically, they asked a pair of strangers to play a novel sequential dyadic die-rolling task together. Each of them privately rolled a die and report her/his outcome in turn; they could maximize payoffs by reporting an identical outcome regardless of its low probability (~16.7%). Using this paradigm, Weisel and Shalvi (2015) have found that participants reported dishonestly more frequently than the chance level, that collaborative settings intensified this tendency compared with situations in which participants played alone, and that the impact of collaboration on promoting corrupt behavior was not modulated by the incentive structure of the task. Most importantly, participants were willing to lie even when only their partners could profit from it, indicating that their willingness to engage in corrupt collaboration could not be accounted for by cost-benefit calculation (Weisel and Shalvi 2015). The robustness of these findings has been verified by follow-up studies (e.g. Wouda et al. 2017; Leib et al. 2021; Spadaro et al. 2022). However, the specific roles of the honesty and reciprocity norms in the establishment of corrupt collaboration remain unclear. In order to fully comprehend the conflict between these 2 social norms during corrupt collaboration as an interpersonal dynamic, it would be meaningful to track the mind of all the individuals being involved and their interactions—more specifically, to examine whether and how the individuals who favor the honesty/reciprocity norm change their mind under the influence of those who favor the other norm.

For this purpose, the current study employed the hyperscanning approach, that is, measuring multiple participants’ brain activation simultaneously to unravel the brain–behavior–brain relationship, moving beyond single-subject observation (Dumas et al. 2011; Hasson et al. 2012; Konvalinka and Roepstorff 2012; Babiloni and Astolfi 2014). Previous hyperscanning research has advanced our understanding of social decision-making (including cooperation and deception) by emphasizing the significance of interbrain neural synchronization in brain regions involved in reward processing and social learning (King-Casas et al. 2005; Tomlin et al. 2006; Jahng et al. 2017; Tang et al. 2019; Zhang et al. 2019; Chen et al. 2020; Pan et al. 2023). Our hyperscanning study was based on functional near-infrared spectroscopy (fNIRS), a noninvasive brain imaging technique for detecting cortical activity (Lu et al. 2010; Cui et al. 2012). In consideration of our research interest and the technical limitations of fNIRS (Huo et al. 2021), our data analysis focused on 2 specific brain regions, that is, the dorsolateral prefrontal cortex (dlPFC) and temporal–parietal junction (TPJ). The dlPFC is closely associated with cognitive control to overcome inappropriate behavior (Speer et al. 2022). The activation level of this region has been found to be negatively correlated with dishonesty (Carlson and Crockett 2018; Yin and Weber 2019; Qu et al. 2020). Indeed, Marechal et al. (2017) discovered that participants’ cheating behavior significantly decreased after the neural excitability of their dlPFC was enhanced using brain stimulation (see also Hu et al. 2022). Additionally, dlPFC lesions reduce honesty concerns in economic games, indicating that this region is necessary for maintaining the honesty norm over self-interested motives (Zhu et al. 2014). In line with this interpretation, a recent study demonstrated that dlPFC synchronization predicted the invested amount to one’s partner in the trust game, suggesting that participants who exhibited higher levels of dlPFC synchronization were more confident in their partners’ honesty (Cheng et al. 2022). Meanwhile, the TPJ (as a key node of the “social brain”) is heavily involved in other-regarding considerations (Morishima et al. 2012; Tusche et al. 2016; Liu et al. 2020). Structural and functional properties of the TPJ are positively correlated with the concern for other people’s benefit and well-being (Carter et al. 2012; Strombach et al. 2015; Park et al. 2017). Most relevantly, TPJ synchronization between brains has been frequently observed in previous studies investigating cooperation, which signals a shared intention to follow the reciprocity norm (Tang et al. 2016; Xue et al. 2018; Abe et al. 2019; Lu et al. 2019). Accordingly, we suggest that the activities of the dlPFC and TPJ represent the influence of the honesty and the reciprocity norm, respectively.

In addition, this study takes personality characteristics into account. Specifically, we measured the participants’ levels of moral judgment ability and empathic concern (EC) with self-report questionnaires. The association between moral judgment ability and honesty is straightforward (Thoma et al. 1991). Meanwhile, EC refers to a concerned, sympathetic, or compassionate reaction to the need of others, which is an other-oriented factor that affects prosocial behavior (Wilhelm and Bekkers 2010; Chierchia and Singer 2017). People with stronger EC care more about others’ benefit (FeldmanHall et al. 2015; Hu et al. 2017) and are more willing to cooperate with others (Batson and Ahmad 2009; Chierchia and Singer 2017). We predicted that a higher level of moral judgment ability and EC would be related to a stronger tendency to follow the honesty and the reciprocity norm, respectively.

In the current study, we combined the classic experimental paradigm developed by Weisel and Shalvi (2015) with fNIRS hyperscanning. Specifically, a group of 2 players individually reported their die-rolling outcome. When both of them dishonestly reported a favorable outcome to maximize economic benefits, we determined that a corrupt collaboration was established. We predicted that this phenomenon could be observed in the behavioral data. More importantly, we aimed to investigate the influence of the honesty and reciprocity norms on corrupt collaboration in dyads, possibly manifesting as interbrain synchronization in the dlPFC and TPJ, respectively. We also predicted that this influence would be sensitive to participants’ level of moral judgment ability and EC.

## Materials and methods

### Participants

Eighty-six healthy college students (42 females) that aged 20.1 ± 1.9 years (mean ± standard deviation [SD]) were recruited from Sichuan Normal University as paid participants. This sample size is comparable to, or larger than, that of many other fNIRS hyperscanning studies (Cui et al. 2012; Tang et al. 2016; Chen et al. 2020; Pan et al. 2023). All participants were right-handed and had normal or corrected-to-normal vision. Written informed consent was obtained prior to the experiment. The experimental protocol was approved by the Ethics Committee of Sichuan Normal University.

### Experimental procedure

Prior to the experiment, participants were required to fill out 2 questionnaires. The first one is the Moral Judgment Test (MJT), which is used to measure an individual’s moral judgment ability (Lind 1978). This questionnaire scores from 0 to 100, with 1–9, 10–29, and >30 indicating low, median, and high moral judgment competence, respectively. The second one is the Interpersonal Reactivity Index (IRI: Davis 1983), which defines empathy as the reactions of one individual to her/his observed experiences of another. The measure has 4 subscales, that is, perspective taking, fantasy, EC, and personal distress. This study used the subscale of EC, which assesses “other-oriented” feelings of sympathy and concern for unfortunate others, in light of previous literature linking this dimension with interpersonal trust and altruistic behavior (e.g. FeldmanHall et al. 2015). The EC subscale includes 7 items and scores from 0 to 28, with a higher score denoting a higher level of EC.

The experiment used the sequential dyadic die-rolling paradigm designed by Weisel and Shalvi (2015) with only 1 modification. That is, the die-roll game used by Weisel and Shalvi (2015) was replaced by a coin-toss game to increase the ecological validity of the task, considering that many Chinese students might not be familiar with die-roll gambling in their daily lives. Participants were randomly paired with a same-gender unacquainted partner. During the formal experiment, the paired participants sat on opposite sides of a table, each facing a computer screen. Stimulus presentation and behavioral data collection were conducted using E-prime program (v3, Psychology Software Tools, Inc., Pittsburgh, USA). Neither verbal nor nonverbal communication was allowed. Before the task, the paired participants were randomly labeled as players A and B. Then they finished a practice session of 10 trials to ensure that they had fully understood the rules. They were also informed that their final payoff depended on the total score accumulated during the task.

**Fig. 1:**
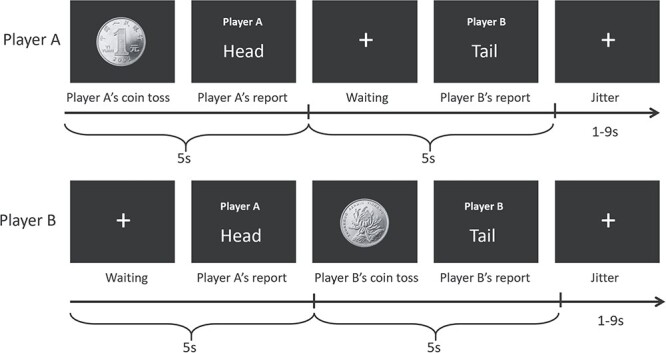
Illustration of the sequential dyadic coin-toss task.

Our task consisted of 3 blocks (40 trials in each) separated by self-paced rest periods. During the task, 2 players individually tossed a coin and reported the outcome. Specifically, player A first “tossed a coin” by pressing the space bar on a keyboard, then reported her/his outcome within 5 s by pressing D or F (indicating “head” or “tail”). After player B received the report from A, she/he also tossed a coin and reported the outcome within 5 s by pressing J or K. Here, different response buttons were assigned to players A and B, in order to facilitate the researchers to distinguish between 2 players’ behavioral responses from the raw data. The sequence of events in an exemplar trial is illustrated in Fig. 1.

Prior to the task, the participants were instructed that each coin-toss would finish privately. Each pair of participants’ reported outcomes determined their payoff. Specifically, if both players reported that the outcome of coin-toss was a head, each of them would earn 2 RMB yuan (~0.3 US dollars); if both reported that the outcome was a tail, each would earn 1 RMB yuan; in cases they reported different outcomes, neither would earn any reward. Given that player B reported her/his outcome after observing player A’s report, she/he had the chance to intentionally match up that report, allowing for corrupt collaboration in this scenario. This game design is in line with the classic task design by Weisel and Shalvi (2015).

The actual outcome of coin-toss was controlled by our E-prime program, such that the probabilities of getting a head and a tail were both 50%. The meanings (head/tail) of the buttons for outcome reporting were counter-balanced across different pairs of participants. The inter-trial interval was randomized from 1 to 9 s. After the experiment, the participants were debriefed and received remuneration based on their game performance.

### fNIRS data recording

**Fig. 2:**
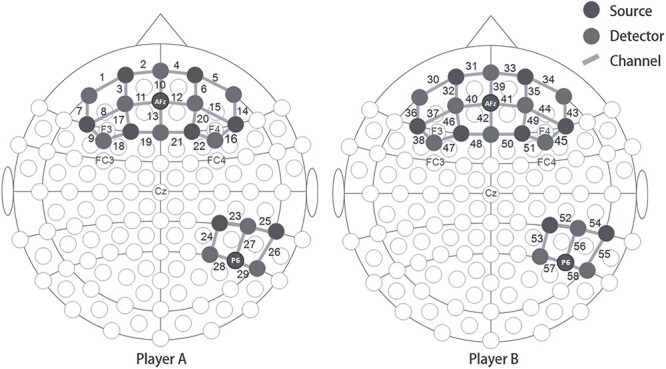
The placement of NIRS optodes and channels.

NIRS data were recorded in a continuous-wave mode using the NirScan system (Danyang Huichuang Medical Equipment, Danyang, China), which consisted of 10 LED emitters (mean intensity = 2 mW/wavelength) and 11 detectors at 2 wavelengths (760 and 850 nm). Optodes were placed to cover the frontal and right temporoparietal cortical regions (Cui et al. 2012; Gradin et al. 2016), using a NIRS-EEG compatible cap (EASYCAP, Herrsching, Germany) in accordance with the international 10/10 system. For each participant, there were 22 valid channels in the frontal area and 7 valid channels in the right temporoparietal region (Fig. 2). Optical sources and detectors were at a mean distance of 3.2 cm (ranging from 2.8 to 3.6 cm). The data were recorded continuously at a sampling rate of 21 Hz. The data recording of 3 pairs of participants encountered some technical problems, resulting in missing NIRS data for 1 player in 2 pairs and missing behavioral data for 1 player in 1 pair. Consequently, the final sample included 40 pairs of NIRS data for analyses.

NIRS channel locations were defined as the central zone of the light path between each adjacent source-detector pair. A toolbox NFRI (http://brain.job.affrc.go.jp/tools/; see Singh et al. 2005) was used to estimate the NMI coordinates of the channel center and transform the coordinates to brain labels according to Brodmann Talairach atlas (Lancaster et al. 2000; Tzourio-Mazoyer et al. 2002) and LPBA40 atlas (Shattuck et al. 2008). Spatial registration of each channel is shown in Supplementary Table S1 (Supplementary Material: Section 1).

### fNIRS data processing

The data were processed using MATLAB R2017b (MathWorks, Natick, MA, USA). NIRS data were screened manually; channels with detector saturation were removed. Intensity data were then converted into optical density changes (ΔOD), followed by artifact correction using spline interpolation. The cleared ΔOD data were filtered using a band-pass filter (0.01–0.2 Hz). Finally, the ΔOD of both measured wavelengths were transformed into relative concentration changes of oxyhemoglobin and deoxyhemoglobin (Δ[HbO] and Δ[Hb]) based on the modified Beer–Lambert law (Cope and Delpy 1988). The differential path length factor was assumed to be 6 for both wavelengths. Although both Δ[HbO] and Δ[Hb] were derived, we selectively performed statistical analyses on Δ[HbO] because of its superior sensitivity in the evaluation of functional activity across conditions (Cui et al. 2011).

As mentioned in the Introduction, this study focused on 2 regions of interests (ROIs), namely, the right TPJ (Brodmann area [BA] 39 and 40) and right dlPFC (BA 8, 9, 10, and 46). We focused on the right part of these 2 regions because previous studies have indicated that the associations between them and social behavior tend to be right lateralized (Knoch et al. 2006; Yang et al. 2020). According to the placement (Fig. 2) and spatial registration of NIRS channels (Supplementary Table S1), the following channels were included in the ROIs (taking player A as an example): channels 15, 16, 20, and 22 for the right dlPFC and channels 23, 24, 27, and 28 for the right TPJ. For the interbrain synchronization, Pearson correlation coefficient *r* was calculated between the 2 10-s epoch of Δ[HbO] data collected from the 2 players in each trial (Dai et al. 2018). Then, the obtained *r* values on trial level were averaged across trials according to different experimental conditions. The measurements of interbrain synchronization (*r* value) were first calculated in each NIRS channel, followed by averaging across channels within the 2 ROIs (Dai et al. 2018).

### Analysis of brain–behavior relationship

Besides interbrain synchronization, we also examined the relationship between interbrain synchronization and behavioral outcomes, as well as the potential mediating effect of interbrain synchronization between participants’ moral/empathic levels and corrupt cooperation. First, a multiple linear regression model was built (enter method) with corrupt cooperation behavior (measured as the times of corrupt cooperation in 120 trials) as the dependent variable. The 4 predictors of the model were the interbrain synchronization of the (i) right dlPFC during honest cooperation, (ii) right dlPFC during corrupt cooperation, (iii) right TPJ during honest cooperation, and (iv) right TPJ during corrupt cooperation.

Second, mediation analysis was performed to examine the mediation role of interbrain synchronization on the relationship between participants’ moral/empathic abilities and corrupt cooperation. In light of the significant correlation between the cheating rate and player A’s moral judgment ability and that between the cheating rate and player B’s EC, we examined (i) the mediating effect of the interbrain dlPFC synchronization during honest cooperation between player A’s moral score and corrupt cooperation and (ii) the mediating effect of the interbrain TPJ synchronization during corrupt cooperation between player B’s empathy score and corrupt cooperation. These effects were examined by the SPSS version of the PROCESS macro based on 1,000 bootstraps resamples and were considered statistically significant when the 95% confidence intervals (CIs) did not include 0 (Hayes 2013).

### Statistics

The significance level was set at 0.05. Descriptive measures were reported as mean ± SD, unless otherwise specified. Regarding interbrain synchronization, paired samples *t*-test (2-tailed) was conducted between conditions and permutation test was used to control the interbrain effect accounted for by “condition similarity” (Supplementary Material: Section 2). Bonferroni correction for multiple comparisons was performed across: (i) within-subject conditions, (ii) ROIs, and (iii) multiple correlation analyses between behavioral/neural indexes and moral/empathy scores.

## Results

### Behavioral results

**Fig. 3:**
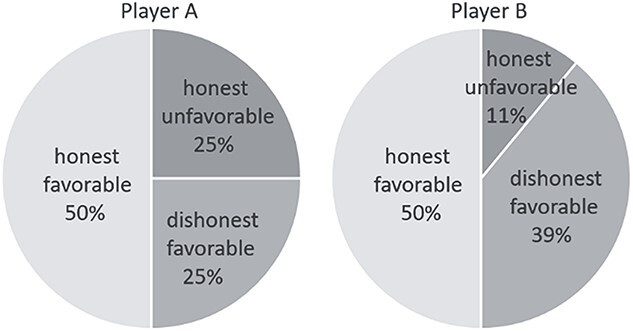
Percentage of reported outcomes. Honest favorable: honestly reported a favorable outcome; honest unfavorable: honestly reported an unfavorable outcome; dishonest favorable: dishonestly reported a favorable outcome.

**Fig. 4:**
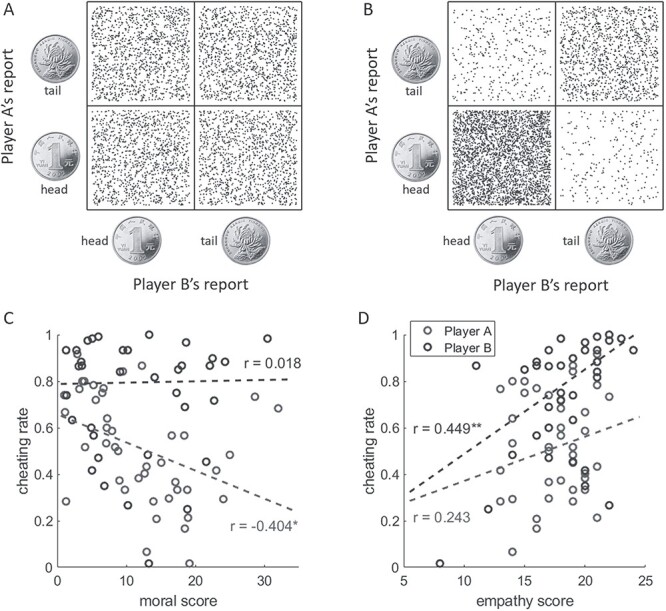
Participants’ reports and their associations with morality and empathy. A) Simulated outcomes assuming honest reporting. B) Real reported outcomes. The positions of dots in A) and B) are jittered to allow visibility. Dot number = 40 pairs of participants × 120 trials = 4,800. C) Correlation between the cheating rate and moral level. D) Correlation between the cheating rate and empathy level. ^*^P < 0.05, ^*^^*^P < 0.01.

Average trial numbers per condition across 120 trials are as follows. Player A honestly reported a favorable outcome (head) in 60.0 ± 9.1 trials, honestly reported an unfavorable outcome (tail) in 30.0 ± 14.7 trials, and dishonestly reported a favorable outcome in 30.0 ± 14.8 trials (Fig. 3A, left pie). Player B honestly reported a favorable outcome (i.e. the same with player A’s outcome) in 60.0 ± 5.3 trials, honestly reported an unfavorable outcome (different from player A’s outcome) in 13.2 ± 15.0 trials, and dishonestly reported a favorable outcome in 46.8 ± 15.8 trials (Fig. 3A, right pie). Paired samples *t*-test showed that player B dishonestly reported a favorable outcome more frequently than player A (*t*(42) = 5.2, *P* < 0.001). Player B’s detailed behavioral data are reported in Supplementary Material (Section 3). Compared with the distribution of the simulation data that were generated based on the assumption that the 2 players always honestly reported the real outcome of coin-toss (Fig. 4A), the distribution of actual behavioral data (Fig. 4B) indicated that corrupt cooperation existed (Weisel and Shalvi 2015).

According to self-reports, we calculated the cheating rate by dividing the number of dishonestly reporting a favorable outcome by the number of actual unfavorable outcome trials. The actual favorable outcome trials were not taken into account, as the cheating rate in those trials was close to 0 across individuals. The results showed an average cheating rate of 50.0 ± 24.7% for player A and 78.0 ± 25.3% for player B. Follow-up analyses confirmed that the cheating rate increased linearly across 3 blocks for player B (*F*(1, 76)_adjusted_ = 4.0, *p*_adjusted_ = 0.049, *η*^2^*_p_* = 0.029), but not for player A (*F*(1, 126) = 1.085, *P* = 0.300, *η*^2^*_p_* = 0.009; see Supplementary Fig. S2 in Supplementary Material, Section 4). Correlation analyses were then performed between the cheating rate and self-reported moral judgment ability (measured by the MJT) as well as EC (measured by the EC subscale of IRI). There was a negative correlation between player A’s cheating rate and her/his moral judgment ability (*r* = −0.40, *P* = 0.007, corrected *P* = 0.014, Fig. 4C). Meanwhile, there was a positive correlation between player B’s cheating rate and her/his EC (*r* = 0.45, *P* = 0.003, corrected *P* = 0.006; Fig. 4D).

At the inter-individual level, considering both players A and B’s intention (honest/dishonest) and the actual outcome (favorable/unfavorable) would lead to 9 conditions (3 × 3) in a dyad, with the trial numbers being hugely imbalanced across conditions (ranging from 3 to 45 trials). For the interest of this study, we focused on 2 conditions: honest cooperation (i.e. both players honestly reported the same outcome: 41.3 ± 9.4 trials per pair, 34.4% out of the total trials) and corrupt cooperation (i.e. both players dishonestly reported the same outcome: 13.6 ± 9.6 trials per pair, 11.4% out of the total trials).

### Interbrain synchronization

As mentioned above, this section focused on the interbrain synchronization of 2 conditions, that is, honest cooperation (41 trials per pair on average) and corrupt cooperation (14 trials per pair on average). The synchronization level of these 2 conditions (indexed by Pearson correlation) was first compared with baseline (the resting period before the whole task): paired samples *t*-tests revealed that the *r* values of the dlPFC (honest: 0.191 ± 0.152, *t*(39) = 6.0, *P* < 0.001; corrupt: 0.130 ± 0.072, *t*(39) = 6.4, *P* < 0.001) and the TPJ (honest: 0.122 ± 0.090, *t*(39) = 4.7, *P* < 0.001; corrupt: 0.219 ± 0.095, *t*(39) = 10.2, *P* < 0.001) were both significantly larger than the interbrain synchronization during resting state (dlPFC: 0.045 ± 0.048; TPJ: 0.051 ± 0.033).

Then permutation tests were performed to discriminate between “mindset synchrony” and “condition similarity” (Supplementary Material: Section 2). Specifically, we randomly assigned 1 player A and 1 player B into a pseudo pair to “reconstruct” the 40 pairs in the whole sample; this random-assignment process was repeated for 500 times to calculate the 95% CI of mean *r* across 40 pairs for the null hypothesis. The mean *r* values of the dlPFC (0.191) fell outside of the 95% CI of the null-hypothesis distribution in the honest [0.043 0.155] (but not corrupt: 0.130 was within [0.038 0.144]) cooperation condition. Meanwhile, the mean *r* values of the TPJ (0.219) fell outside of the 95% CI of the null-hypothesis distribution in the corrupt [0.062 0.172] (but not honest: 0.122 was within [0.037 0.140]) cooperation condition. Accordingly, we conclude that the increased synchronization was resulted from mindset synchrony (rather than condition similarity) between 2 interactive brains at the dlPFC during honest cooperation and at the TPJ during corrupt cooperation, at a significance level of *P* < 0.05.

**Fig. 5:**
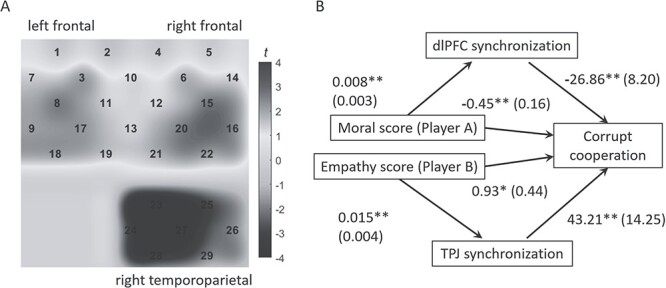
Interbrain synchronization and its influence on corrupt cooperation. a) The *t*-value map of the correlation *r* comparing between *honest cooperation* vs. *corrupt cooperation* conditions. The channel numbers are illustrated. b) Mediating effect of interbrain synchronization between moral/empathy scores and corrupt cooperation. Unstandardized coefficients are shown as means (SE). Statistically significant pathways are indicated using solid lines. ^*^P < 0.05, ^*^^*^P < 0.01.

Finally, we compared the brain synchronization between honest and corrupt cooperation conditions at the dlPFC and TPJ (Fig. 5A). Paired samples *t*-test shows that the brain synchronization significantly increased during honest cooperation compared with corrupt cooperation at the right dlPFC (*t*(39) = 2.5, *P* = 0.018, corrected *P* = 0.036), whereas the brain synchronization significantly increased during corrupt cooperation compared with honest cooperation at the right TPJ (*t*(39) = −5.0, *P* < 0.001, corrected *P* = 0.001).

### Brain–behavior relationship

In this section, we reported the relationship between interbrain synchronization and behavioral outcomes, as well as the potential mediating effect of interbrain synchronization between participants’ moral/empathic levels and corrupt cooperation.

First, a multiple linear regression model was built (enter method) with corrupt cooperation behavior (measured as the times of corrupt cooperation in 120 trials) as the dependent variable. The 4 predictors of the model were the interbrain synchronization of the (i) right dlPFC during honest cooperation, (ii) right dlPFC during corrupt cooperation, (iii) right TPJ during honest cooperation, and (iv) right TPJ during corrupt cooperation. Results show that the interbrain dlPFC synchronization during honest cooperation (standardized coefficient = −0.42, *t* = −2.9, *P* = 0.007) and the interbrain TPJ synchronization during corrupt cooperation (standardized coefficient = 0.35, *t* = 2.4, *P* = 0.020) significantly predicted corrupt cooperation at the behavioral level (*R*^2^ = 0.47, *F*(4, 35) = 7.6, *P* < 0.001). That is to say, a stronger interbrain TPJ synchronization during corrupt cooperation was associated with more corrupt behaviors (*r* = 0.58, *P* < 0.001), whereas a stronger interbrain dlPFC synchronization during honest cooperation was associated with fewer corrupt behaviors (*r* = −0.59, *P* < 0.001).

Second, mediation analysis was performed to examine the mediation role of interbrain synchronization on the relationship between participants’ moral/empathic abilities and corrupt cooperation. In light of the significant correlation between the cheating rate and player A’s moral judgment ability (Fig. 4C) and that between the cheating rate and player B’s EC (Fig. 4D), we examined (i) the mediating effect of the interbrain dlPFC synchronization during honest cooperation between player A’s moral score and corrupt cooperation and (ii) the mediating effect of the interbrain TPJ synchronization during corrupt cooperation between player B’s empathy score and corrupt cooperation. These effects were examined by the SPSS version of the PROCESS macro based on 1,000 bootstraps resamples and were considered statistically significant when the 95% CIs did not include 0 (Hayes 2013).

Results showed that the indirect effect of player A’s moral judgment ability on corrupt cooperation via interbrain dlPFC synchronization was significant (*B* = 0.008 × −26.86 = −0.21, SE = 0.09, 95% CI = [−0.43 to −0.06]). This mediation model accounted for 32.9% of the total effect from player A’s moral score to corrupt cooperation. Since the direct effect of player A’s moral judgment ability on corrupt cooperation was still significant after controlling the impact of interbrain dlPFC synchronization, we determined that interbrain dlPFC synchronization exhibited a partial mediating effect (Fig. 5B, top panel). Meanwhile, the indirect effect of player B’s EC on corrupt cooperation via interbrain TPJ synchronization was significant (*B* = 0.015 × 43.21 = 0.65, SE = 0.38, 95% CI = [0.04–1.52]). This mediation model accounted for 40.4% of the total effect from player B’s empathy score to corrupt cooperation. Since the direct effect of player B’s EC on corrupt cooperation was still significant after controlling the impact of interbrain TPJ synchronization, we determined that the interbrain TPJ synchronization exhibited a partial mediating effect (Fig. 5B, bottom panel).

## Discussion

Regardless of the significance of corrupt collaboration in modern-day society, little is known about the motivational basis of this kind of illegal and unethical behavior (Jávor and Jancsics 2016). Researchers have recently proposed that corrupt collaboration arises when the honesty norm and the reciprocity norm clash during interpersonal interaction (Weisel and Shalvi 2022). To examine this possibility, and to investigate the potential role of personality variables in the conflict between different social norms, the current study combined a well-validated behavioral task designed by Weisel and Shalvi (2015) and fNIRS hyperscanning—a technique that facilitates the investigations on dyadic social interactions (Nguyen et al. 2020). Our behavioral results confirmed previous findings that corrupt collaboration could be observed in this task, that is, 2 interacting players dishonestly (or we may call “corruptly”) reported the same coin-toss outcome in a large percentage of trials (Weisel and Shalvi 2015; Wouda et al. 2017; see also Spadaro et al. 2022). Furthermore, players A and B displayed diverse behavioral patterns and were influenced differently by their moral judgment ability and EC levels, indicating that the honesty and reciprocity norms affected individual corrupt behavior (indexed by the cheating rate) in different ways. Most notably, the fNIRS results improve the knowledge of the above behavioral phenomena, such that the interbrain synchronization in specific regions (including the dlPFC and TPJ) was not only correlated with corrupt collaboration, but also mediated the relationship between moral judgment ability/EC and corrupt collaboration.

According to the task design from Weisel and Shalvi (2015), player A independently determines whether to dishonestly report the die-rolling outcome (i.e. the one who “sets the stage”), whereas player B observes A’s report and then decides whether to match that report (i.e. the one who “gets the job done”). This nonsymmetrical situation mimics real-life scenarios where individuals often play different roles in corrupt collaboration. For instance, an employee who initially shows no interest in corrupt practices may be swayed to participate after witnessing the corrupt behavior of their colleagues (Chang and Lai 2002; Ashforth et al. 2008). In Weisel and Shalvi’s (2015) task, players were unable to know if their partner’s report was dishonest in any single trial, but we believe that player B could have noticed that A frequently reported a favorable outcome (~75% of the trials) and interpreted this as an “invitation to lie” signal for group-serving dishonesty (see also Weisel and Shalvi 2015). In line with this idea, player B’s corrupt behavior gradually increased over the 3 blocks, but player A showed no such trend; that is to say, player B was corrupted through her/his interactions with player A (see also Moore 2009). Accordingly, we suggest that player B was more greatly influenced by the reciprocity norm compared with player A.

Our understanding of the aforementioned results is further deepened by the relationship between player A or B’s decisions and their level of moral judgment ability or EC. Specifically, player B’s EC score was positively correlated with her/his corrupt behavior. We believe that when player B had a higher level of EC, she/he would care more for player A’s benefit and thus were more willing to match A’s report for maximizing the collective income. From this perspective, our behavioral results support the notion that corrupt collaboration may stem from prosocial motives, as suggested by Weisel and Shalvi (2022). Conversely, if self-interest was player B’s only driving force, empathy should have no role in corrupt collaboration. Meanwhile, player A’s corrupt behavior was negatively correlated with her/his moral judgment ability (see also Marechal et al. 2017). While player B could point to benefiting player A to justify corrupt practices (see also Conrads et al. 2013; Gino et al. 2013), player A made the decision independently in each trial and had to take full moral responsibility for that decision. Consequently, when her/his level of moral judgment ability was higher, player A would be more likely to feel an obligation to follow the honesty norm, even though dishonesty could not be detected in the task (see also Mazar et al. 2008; Fischbacher and Föllmi-Heusi 2013). In short, the influences of the honesty and reciprocity norms on corrupt collaboration manifested as the effects of the moral judgment ability and empathy of the participants, respectively. Additionally, these factors can differ based on the social roles of the individuals involved in the situation, such as the initiator or finalizer of a corrupt cooperation.

Our fNIRS hyperscanning data may shed light on the interpersonal communication processes involved in the establishment of corrupt collaboration (see also Hasson et al. 2012). Taking both the relevant literature (e.g. Cui et al. 2012; Cheng et al. 2015; Balconi et al. 2017; Chen et al. 2020) and technical limitations (see Lloyd-Fox et al. 2010, for details; Ferrari and Quaresima 2012) into account, our fNIRS recording focused on 2 selected ROIs including the right dlPFC and TPJ. The major findings are as follows. First, the right dlPFC synchronization became stronger for the honest cooperation condition than the corrupt cooperation condition, whereas the reverse was true regarding the right TPJ synchronization. Second, the right dlPFC synchronization under the honest cooperation condition was negatively correlated with corrupt collaboration, whereas the right TPJ synchronization under the corrupt cooperation condition was positively correlated with corrupt collaboration. Finally, the right dlPFC synchronization partly mediated the relationship between player A’s moral judgment ability and corrupt collaboration, whereas the right TPJ synchronization partly mediated the relationship between player B’s EC and corrupt collaboration. Additionally, our permutation tests confirmed that these findings could not be accounted for by “condition similarity,” that is, the same task settings and experimental environment across all participants (Hasson et al. 2012; Burgess 2013; Zhang et al. 2019); instead, they should be explained according to the shared opinions and beliefs between a dyad, that is, “mindset synchrony” (Zhang et al. 2019). In our opinion, the aforementioned between-subject synchronizations help interpret how individuals adhering to the honesty (or reciprocity) norm influence others to avoid (or participate in) corrupt collaboration.

On one hand, previous literature has associated the dlPFC with honesty, as we mentioned in the Introduction. For instance, Hu et al. (2022) discovered that perturbing the right dlPFC via brain stimulation strengthened participants’ willingness to engage in corrupt behavior, such as taking more bribes (see also Hu et al. 2021). In line with our results, the importance of the dlPFC synchronization in honesty and interpersonal trust has been discovered in recent research (Nguyen et al. 2021; Cheng et al. 2022). Furthermore, we found that the dlPFC synchronization mediated the relationship between player A’s moral responsibility level and corrupt collaboration. As we mentioned earlier, player A took full moral responsibility for her/his decision because of the game rules, which may have subjected her/him to higher moral pressure for dishonest reports compared with player B. We believe that for player A, a greater sense of moral responsibility resulted in stronger adherence to the honesty norm as reflected by her/his decisions, which then influenced player B’s mind (manifesting as an increased dlPFC synchronization within a dyad).

On the other hand, previous research has linked the right TPJ synchronization with a shared motivation to cooperate between individuals (Era et al. 2020; Shiraishi and Shimada 2021). It is important to note that in this study, the TPJ synchronization was enhanced for corrupt cooperation than honest cooperation. In our view, this was partly because honest cooperation could be driven by individual willingness of following the honesty norm rather than by a shared cooperative motivation. In addition, the relationship between the TPJ and mentalizing capacity (i.e. the ability to perceive other’s intentions and feelings) might be crucial in explaining our results (Adolphs 2009; Frith and Frith 2012). Numerous studies have demonstrated that the right TPJ subserves the mentalizing process during social communication (Harada et al. 2009; Lisofsky et al. 2014; Suchotzki et al. 2015). Based on this understanding, we propose that the synchronized activity in the right TPJ associated with corrupt cooperation indicated that a pair of players had successfully detected each other’s intention to manipulate the coin-toss outcome report. This mutual recognition of cooperative intentions reinforced their corrupt collaboration, mimicking what often occurs in real-life scenarios (Li 2018). Furthermore, previous studies have revealed that individuals with a higher level of EC are more capable of detecting others’ intentions (Kaplan and Iacoboni 2006; Li et al. 2015; Baez et al. 2016). Thus, it is not surprising that the TPJ synchronization mediated the relationship between player B’s EC level and corrupt collaboration. That is, when player B had a higher level of EC, she/her was better able to detect her/his partner’s intention to maximize economic benefit, and conformed to that intention according to the reciprocity norm. We suggest that our TPJ findings would provide insights into the well-known “bad barrel” effect, which refers to the phenomenon that individuals can be influenced by corrupt organizations to engage in unethical behavior (Ashforth et al. 2008; Jávor and Jancsics 2016).

To summarize, the current findings indicate the following: (i) conforming to the reciprocity norm motivates people to participate in corrupt collaboration, whereas the honesty norm has the opposite effect (see also Weisel and Shalvi 2022), (ii) the influence of the honesty and reciprocity norms is modulated by individual level of moral judgment ability and EC, respectively, (iii) in asymmetrical social dilemmas, people making independent decisions are more strongly influenced by the honesty norm, whereas those observing others’ decisions first are more strongly influenced by the reciprocity norm, and (iv) these 2 influences are manifested as increased synchronization in the dlPFC and TPJ regions, respectively, during social interactions. The current findings not only contribute to our knowledge of the mechanisms behind people’s trade-off between keeping honest and joining forces in corrupt collaboration, but also highlight the situational and dispositional factors that influence this trade-off.

In our opinion, the implications of these findings are multi-faced. First, they confirm the idea from Weisel and Shalvi (2022) that corrupt collaboration reveals a dark side of the reciprocity norm. Reciprocal relationships in the workplace can result in a loosening of moral restrictions, leading people to prioritize their colleagues’ benefits over those of the organization (Ashforth et al. 2008; Tavits 2010). Meanwhile, the honesty norm can be relied upon for anti-corruption, but its effectiveness varies based on situational and personal variables (see also Chang and Lai 2002; Muthukrishna et al. 2017). More broadly speaking, these knowledges improve our understanding of how people resolve social dilemmas with conflicting moral principles (Cialdini et al. 1991; Christensen and Gomila 2012; Ruff et al. 2013; Levine and Schweitzer 2014). Second, the association between EC and corrupt collaboration has not been previously acknowledged in the literature (Trevino 1986; Den Nieuwenboer and Kaptein 2008). EC is a crucial aspect of human life, but its negative aspects warrant further investigation (see also Xu et al. 2009; Cikara et al. 2011). Third, our findings emphasize the significance of the dlPFC and TPJ during the social interaction process underlying corrupt collaboration, suggesting that they could be candidate regions for targeted interventions.

A few experimental methodology issues and future directions should be noted (see also Jenkins et al. 2016; van Dijk and De Dreu 2020). First, although the behavioral paradigm developed by Weisel and Shalvi (2022) has the advantage of focusing on individual willingness to cheat, the generalizability of its results remains to be verified with alternative paradigms. Follow-up studies should also explore whether the current findings apply to other forms of corruption such as bribery, embezzlement, and cronyism (Ashforth et al. 2008). Second, future research could benefit from manipulating situational factors such as the number of interacting partners, seeing that “diffusion of responsibility” (which increases with group size) can modulate the motivation to participate in corrupt collaboration (Conrads et al. 2013; Crockett et al. 2017; Soraperra et al. 2017). Finally, this paper focuses on the importance of the honesty and reciprocity norms in corrupt collaboration, but it is still possible that self-serving evaluation also play a significant role. According to recent research using functional magnetic resonance imaging, deep brain regions such as the ventromedial prefrontal cortex (which has been associated with self-serving evaluation) are involved in corrupt behavior (Hu et al. 2021), but they are beyond the scope of fNIRS recording. Researchers who aim to gain a more comprehensive understanding of corrupt behavior should acknowledge the technical constraints and consider alternative approaches that take into account both within-subject and between-subject levels of whole-brain observation.

## Author contributions

Dandan Zhang (Conceptualization, Formal analysis, Funding acquisition, Investigation, Visualization, Writing—original draft), Shen Zhang (Data curation), Zhen Lei (Writing—review & editing), Yiwei Li (Data curation), Xianchun Li (Writing—review & editing), and Ruolei Gu (Writing—original draft, Writing—review & editing)

## Supplementary Material

Supplementary_material_bhad132

## Data Availability

All the data and code used in this study could be available by contacting the first author, DZ (e-mail: zhangdd05@gmail.com).
