# Supplementary material for: Why people engage in corrupt collaboration: an observation at the multi-brain level
Source: Cereb Cortex. 2023 Apr 20;33(13):8465–76. doi: 10.1093/cercor/bhad132 (PMC10786094; doi:10.1093/cercor/bhad132)
Supplement: Supplementary_material_bhad132 [file supplementary_material_bhad132.docx]

**Zhang et al.**

Why people engage in corrupt collaboration: An observation at the multi-brain level

***Supplementary material***

## Spatial registration of the functional near-infrared spectroscopy (fNIRS) channels used in this study

Table S1. Spatial registration of the 29 fNIRS channels (Brodmann/LPBA40 area)

| Channel # | | MNI | Channel | Brodmann area  (percentage of overlap)^a^ | LPBA40  (percentage of overlap) ^a^ |
| --- | --- | --- | --- | --- | --- |
| Player A | Player B |  |  |  |  |
| 1 | 30 | -34, 63, -8 | Fp1-AF7 | 10 - Frontopolar area (0.70) | L middle frontal gyrus (0.74) |
| 2 | 31 | -12, 71, -5 | Fp1-Fpz | 10 - Frontopolar area (0.80) | L superior frontal gyrus (0.93) |
| 3 | 32 | -23, 68, 2 | Fp1-AF3 | 10 - Frontopolar area (1) | L middle frontal gyrus (0.97) |
| 4 | 33 | 14, 71, -5 | Fp2-Fpz | 10 - Frontopolar area (0.88) | R middle frontal gyrus (1) |
| 5 | 34 | 36, 64, -9 | Fp2-AF8 | 10 - Frontopolar area (0.72) | R inferior frontal gyrus (0.62) |
| 6 | 35 | 26, 68, 2 | Fp2-AF4 | 10 - Frontopolar area (1) | R middle frontal gyrus (1) |
| 7 | 36 | -46, 51, 1 | F5-AF7 | 10 - Frontopolar area (0.53) | L inferior frontal gyrus (0.92) |
| 8 | 37 | -41, 55, 16 | F5-AF3 | 10 - Frontopolar area (0.85) | L middle frontal gyrus (1) |
| 9 | 38 | -48, 35, 25 | F5-FFC3 | 9/46 - Dorsolateral prefrontal cortex (0.86) | L middle frontal gyrus (0.60) |
| 10 | 39 | 2, 69, 11 | AFz-Fpz | 10 - Frontopolar area (1) | R superior frontal gyrus (0.74) |
| 11 | 40 | -15, 66, 23 | AFz-AF3 | 10 - Frontopolar area (1) | L middle frontal gyrus (0.50) |
| 12 | 41 | 17, 67, 24 | AFz-AF4 | 10 - Frontopolar area (1) | R middle frontal gyrus (0.96) |
| 13 | 42 | 2, 56, 38 | AFz-Fz | 9 - Dorsolateral prefrontal cortex (0.96) | R superior frontal gyrus (0.85) |
| 14 | 43 | 48, 51, 2 | F6-AF8 | 47 - Inferior prefrontal gyrus (0.47) | R inferior frontal gyrus (0.96) |
| 15 | 44 | 43, 55, 16 | F6-AF4 | 10 - Frontopolar area (0.93) | R middle frontal gyrus (0.65) |
| 16 | 45 | 50, 35, 26 | F6-FFC4 | 9/46 - Dorsolateral prefrontal cortex (0.86) | R middle frontal gyrus (0.60) |
| 17 | 46 | -26, 56, 30 | F1-AF3 | 10 - Frontopolar area (0.54) | L middle frontal gyrus (1) |
| 18 | 47 | -33, 38, 43 | F1-FFC3 | 8 - Includes Frontal eye fields (0.51) | L middle frontal gyrus (0.60) |
| 19 | 48 | -10, 45, 51 | F1-Fz | 8 - Includes Frontal eye fields (1) | L superior frontal gyrus (1) |
| 20 | 49 | 29, 56, 31 | F2-AF3 | 9 - Dorsolateral prefrontal cortex (0.53) | R middle frontal gyrus (1) |
| 21 | 50 | 13, 45, 51 | F2-Fz | 8 - Includes Frontal eye fields (1) | R superior frontal gyrus (0.98) |
| 22 | 51 | 35, 38, 44 | F2-FFC4 | 8 - Includes Frontal eye fields (0.57) | R middle frontal gyrus (0.72) |
| 23 | 52 | 62, -43, 45 | CP4-CP6 | 40 - Supramarginal gyrus (1) | R supramarginal gyrus (0.52) |
| 24 | 53 | 50, -56, 53 | CP4-P4 | 40 - Supramarginal gyrus (1) | R angular gyrus (1) |
| 25 | 54 | 69, -43, 10 | TP8-CP6 | 22 - Superior Temporal Gyrus (1) | R middle temporal gyrus (0.85) |
| 26 | 55 | 64, -56, 12 | TP8-P8 | 37 - Fusiform gyrus (0.91) | R middle temporal gyrus (0.93) |
| 27 | 56 | 62, -56, 29 | P6-CP6 | 40 - Supramarginal gyrus (0.71) | R angular gyrus (1) |
| 28 | 57 | 51, -68, 40 | P6-P4 | 39 - Angular gyrus (1) | R angular gyrus (1) |
| 29 | 58 | 57, -67, 13 | P6-P8 | 39 - Angular gyrus (0.56) | R middle occipital gyrus (0.92) |

^a^ One NIRS channel may cover several anatomical labels. For the sake of brevity, here we only report the anatomical labels that have the largest overlap with a channel.

## 2. Permutation test

Previous studies have suggested that there are two potential causes of between-brain coherence of neural signals (Burgess, 2013). The first possible cause is “mindset synchrony,” that is, a real inter-brain synchronization. The second possible cause is “condition similarity,” that is, two independent individuals are asked to perform the same task in the same experimental environment, therefore their brains activate in a similar way (Liu & Pelowski, 2014). To exclude the confounding effects of condition similarity, we employed a permutation test that could help examine the reliability of inter-brain synchronization (Zhang et al., 2019). Specifically, we randomly assigned one player A and one player B into a pseudo pair, who actually did not interact in the experiment simultaneously. Then the mean correlation *r* values were calculated for “honest cooperation” and “corruptive cooperation” trials in each of the 40 pseudo pairs and were averaged across the 40 pairs. This procedure of participant permutation was repeated for 500 times to generate a distribution of mean *r* value under the null hypothesis (i.e., condition similarity). If the mean *r* between the real paired participants fell out of the 95% confidence interval of this null-hypothesis distribution, we conclude that the enhanced inter-brain synchronization was due to real inter-brain communication (i.e., mindset synchrony) at a significance level of *p* < 0.05.

## 3. Player B’s detailed behavioral results

Player B honestly reported a head in 45.0 ± 8.2 trials, honestly reported a tail in 9.6 ± 10.1 trials, and dishonestly reported a head in 35.4 ± 13.6 trials, after player A reported a head. Player B honestly reported a head in 3.6 ± 6.8 trials, honestly reported a tail in 15.0 ± 7.3 trials, and dishonestly reported a tail in 11.4 ± 7.0 trials, after player A reported a tail (Figure S1A).


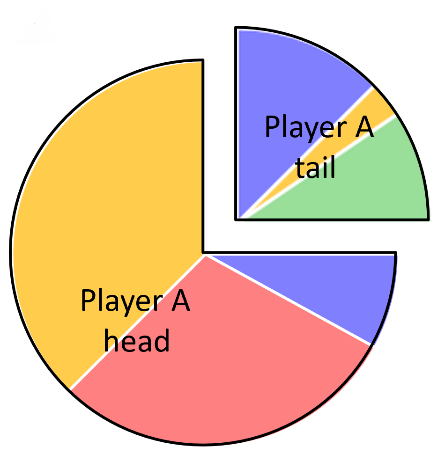


Figure S1. Percent of reported outcomes of Player B.

## 4. Temporal changes of cheating rate

As mentioned in the main text, this study defined the cheating rate as the probability of dishonest reporting trials from the total of unfavorable outcome trials. Here we illustrate the temporal changes of this index by calculating it every 10 trials for player A and player B, respectively (Figure S2A). We analyzed the changes of cheating rate in the three blocks (40 trials per block). First, one-way ANOVA was used to test the cheating rate of the two players across blocks, followed by pairwise comparisons (Figure S2B). For player A, the cheating rate did not change significantly throughout the task (*F*(2,84) = 1.6, *p* = 0.215, *η^2^_p_* = 0.036; block 1, 2, and 3 = 45.3 ± 27.9%, 51.8 ± 27.0%, and 51.8 ± 31.9%, respectively). For player B, the cheating rate increased during the whole task (*F*(2,84) = 10.6, *p* < 0.001, *η^2^_p_* = 0.202), which was higher in block 3 (83.8 ± 21.3%) than block 1 (72.8 ± 29.5%; *p* < 0.001) and block 2 (76.1 ± 28.3%; *p* = 0.017).

We also conducted a linear contrast analysis for player A and player B, respectively. Regarding player A, the linear trend from block 1 to block 3 was insignificant (the assumption of homogeneity of variance was not violated, *p* = 0.339; *F*(1,126) = 1.085, *p* = 0.300, *η^2^_p_* = 0.009). Regarding player B, the linear trend from block 1 to block 3 was significant (the assumption of homogeneity of variance was violated, *p* = 0.029; *F*(1,76)_adjusted_ = 4.0, *p*_adjusted_ = 0.049, *η^2^_p_* = 0.029).


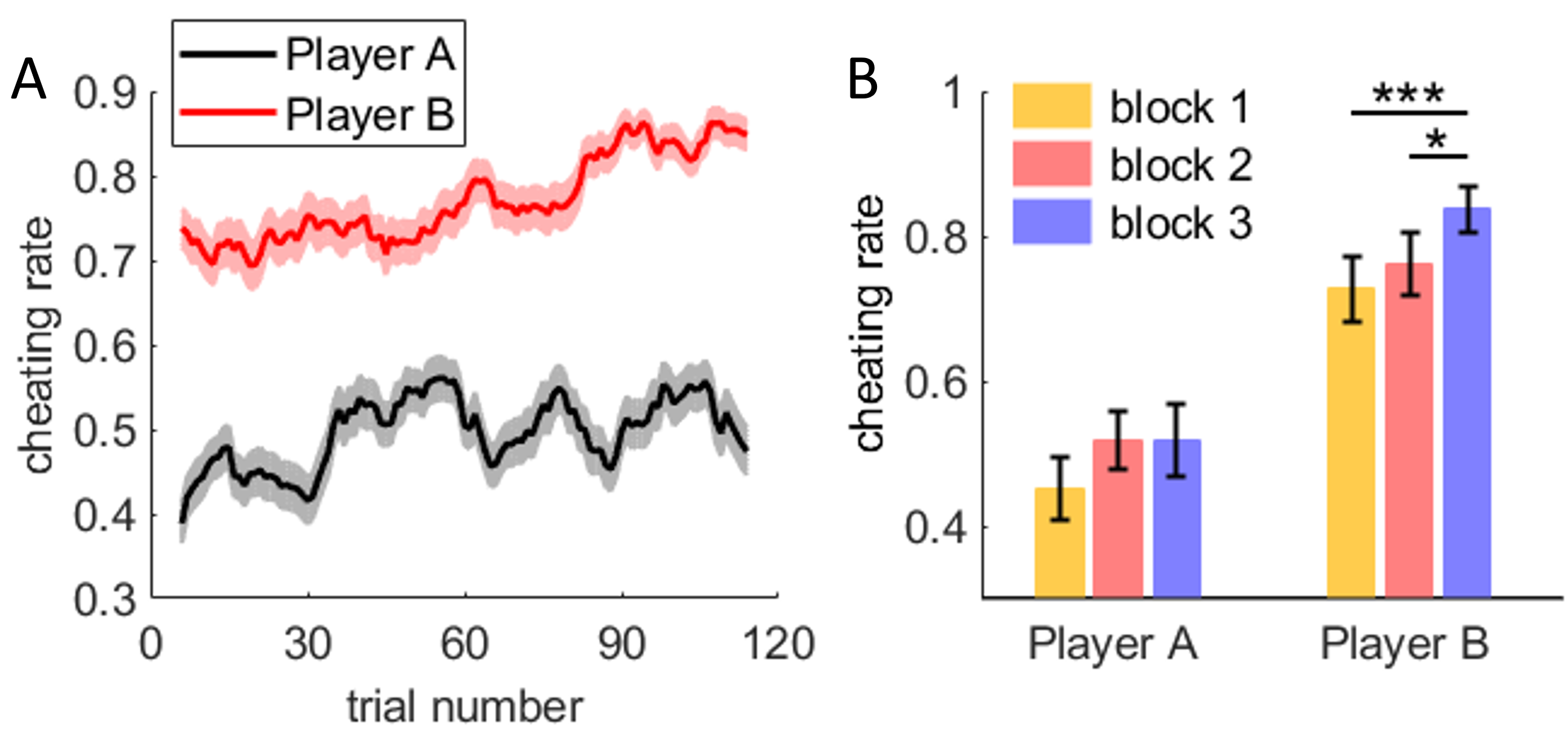


Figure S2. Cheating rate in every 10 trials. A, Temporal waveforms of the cheating rate for player A and player B. Thick lines denote the means while light-colored regions denote standard errors of the mean. B, Cheating rate in the three blocks. Bars represent standard errors of the mean. **p* < 0.05, ****p* < 0.001.

**References**

Burgess, A. P. (2013). On the interpretation of synchronization in EEG hyperscanning studies: A cautionary note. *Frontiers in Human Neuroscience*, *7*, 881. <https://doi.org/10.3389/fnhum.2013.00881>

Liu, T., & Pelowski, M. (2014). Clarifying the interaction types in two-person neuroscience research. *Frontiers in Human Neuroscience*, *8*, 276. <https://doi.org/10.3389/fnhum.2014.00276>

Zhang, D., Lin, Y., Jing, Y., Feng, C., & Gu, R. (2019). The dynamics of belief updating in human cooperation: Findings from inter-brain ERP hyperscanning. *Neuroimage*, *198*, 1-12. <https://doi.org/10.1016/j.neuroimage.2019.05.029>
